# Temporal Dynamics of Reproduction of the Neotropical Fish, *Crenicichla menezesi* (Perciformes: Cichlidae)

**DOI:** 10.1100/2012/579051

**Published:** 2012-08-01

**Authors:** Andréa Soares de Araújo, Wallace Silva do Nascimento, Maria Emília Yamamoto, Sathyabama Chellappa

**Affiliations:** ^1^Programa de Pós-Graduação em Psicobiologia, Departamento de Fisiologia, Centro de Biociências, Universidade Federal do Rio Grande do Norte (UFRN), Avenida Salgado Filho 3000, Lagoa Nova, 59.072-970 Natal, RN, Brazil; ^2^Departamento de Oceanografia e Limnologia, Universidade Federal do Rio Grande do Norte (UFRN), Praia de Mãe Luíza, 59014-100 Natal, RN, Brazil

## Abstract

The reproductive biology and the gonadal development cycle of the Neotropical cichlid fish, *Crenicichla menezesi*, is described. This species exhibits sexual dimorphism only during the spawning season. First sexual maturity of females is attained earlier than the males. Both macroscopic and histological investigations of ovaries and testes revealed four stages of gonadal maturation. Mean batch fecundity of females was 372 (±10,41) of mature oocytes. This species is a partial spawner, with an extended spawning period. Monthly values of GSI and the condition factor are negatively correlated during the gonadal development cycle of this species.

## 1. Introduction

The family Cichlidae represents one of the most diverse groups of freshwater fish in the world [1, 2]. There are about 450 species of Neotropical cichlids distributed widely in Central and South America, especially in the Amazonian basin [2]. Brazil has about 81 species of cichlids which correspond to 6% of total freshwater fish species which are considered economically important. They serve as food for the local population and also have potential for intensive, extensive, and ornamental fish culture [3–5].

The genus *Crenicichla* (Heckel, 1840) has 83 described species and is the second largest genus among the cichlids in South America [3, 6–8]. They are distributed between the regions north of the Amazon River (including Venezuela, Colombia, and Guyana), across the Amazon basin and the Atlantic basins in central and southern Argentina [6]. The two species, *Crenicichla menezesi* and *Cichlasoma orientale*, are considered endemic to the Caatinga ecoregion [9–11]. 

Biological data available on the Neotropical ornamental fish species *C. menezesi* is rather limited. Existing information about its biology has been provided by the ornamental fish culturists [12, 13]. However, the reproductive biology of this species is still not well understood. This paper presents a study which examined the reproductive biology of *C. menezesi*, including the length-weight analysis, size at first sexual maturity, macroscopic and histological descriptions of gonad developmental stages, fecundity, type of spawning, changes in the gonadosomatic index, the condition factor, and the reproductive period in the natural environment. An understanding of its breeding biology will be helpful for the conservation of this species besides developing captive rearing systems for commercial purposes.

## 2. Materials and Methods

### 2.1. Study Area and Sample Collection

 Fish samplings were carried out in the Marechal Dutra Reservoir, located in Northeastern Brazil (6° 26′ 11′′S and 36° 36′ 17′′W). The reservoir has an accumulation capacity of 40 × 10^6^ m^3^ with a maximum depth of 25 m and a mean depth of 8.5 m and was constructed in the hydrographic basin of River Piranhas-Assu, which covers an area of 44000 km². This reservoir is the main source of drinking water for the Acari village, besides being important for fisheries and agriculture. 

Fish samples were captured during March 2009 to February 2010 covering an annual cycle. Fish samples were captured with the help of local fishermen using traditional fish traps, which were circular with 15 cm of diameter and 30 cm in length. The traps made of PVC material were left for 24 hours in the reservoir and were lifted at the end of this period. Fish captured were numbered, measured, and weighed. Samples of fish were used for morphometric measurements and meristic counts to verify the taxonomical identification of the study species. The taxonomical status of this species was confirmed by Professor Dr. Ricardo Souza Rosa, of the Department of Systematics and Ecology of the Federal University of Paraíba, Brazil. Sample specimens of this species were deposited in the museum collection of this institution.

### 2.2. General Biological Measurements

 Measures of total length (Lt ± 1 cm) (distance from the anterior extremity of the maxilla to the final extremity of the tail fin), total body mass (Wt ± 1 g), and gonad weight (Wg ± 0.1 mg) were recorded for each fish separately. Monthly distribution analysis of total length and weight of males and females were performed separately using the absolute frequencies (mean ± SD). The size frequencies were grouped into eight classes for total length (Lt) with intervals of 20 cm and eight classes for total body mass (Wt) with intervals of 10 g. 

### 2.3. Sex Ratio

 The sex ratio was established by the ratio between the number of males and females during the study period, without including the immature individuals [14]. Young individuals were not included as it was not possible to determine their sex by macroscopic observations. Monthly sex ratios were tested using an *χ* ^2^ test to verify significant differences from an expected 1 : 1 ratio, at 5% level of significance. 

### 2.4. Length at First Gonadal Maturity (*L_50_*)

*L*_50_ was defined as the smallest predicted length interval, in which 50% of the individuals were mature. This was estimated from the relative frequency distribution of adult males and females, using their total length classes with intervals of 2 cm [15]. 

### 2.5. Macroscopic and Histological Examinations of Ovaries

The location and general aspects of the ovaries were observed and stages of reproductive maturity were determined using a macroscopic staging system. The ovaries were classified as immature (very small ovaries, translucent, without visible oocytes); maturing (ovaries occupying 25% to 50% of the body cavity, with visible oocytes); mature (ovaries ranging from medium to large, almost occupying 75% of the body cavity with vascularization and with opaque oocytes); spent (flaccid ovaries, ranging from medium to small) [16]. Turgidity, color, and presence of blood vessels of the ovaries were also observed. In order to avoid possible variation in the developmental stage of oocytes due to their position in the ovaries, histological examinations were carried out on sections from the anterior (cephalic), middle (central), and posterior (caudal) regions of 20 ovaries which were in different developmental stages [17]. These data were later compared in order to determine whether samples taken from mid-section of the ovary of either lobe were representative of oocyte development. 

The ovaries and testicles were preserved in Bouin's solution, later embedded in paraffin, sectioned at 3–5 *μ*m thickness, and stained with Harris hematoxylin and eosin (H&E). Gonadal developmental stages were assessed microscopically with the help of light microscope (Taimin, model TM 800), coupled with a video camera (Kodo Digital). Existing terminology was used for staging of oogenesis [18, 19].

### 2.6. Fecundity and Type of Spawning

To determine the fecundity, ovaries were removed from 14 mature females, weighed and preserved in Gilson solution for 24 hours for complete dissociation of oocytes, which were then washed and preserved in 70% ethyl alcohol [20]. A 10% sample was removed for counting of mature oocytes, and the values were extrapolated to 100%. 

The correlations between fecundity × body weight and fecundity × weight of ovaries were verified. The relative frequency of the different diameter sizes of oocytes was estimated by enumerating three subsamples of the different-sized oocytes in each ovary [18]. The diameters of oocytes from all ovaries were measured with an ocular micrometer (Wild M7, objective 31x and ocular 10x). 

Spawning pattern was assessed by oocyte diameter distributions [18]. The oocytes of mature ovaries were grouped into classes of different diameter sizes, based on their frequency of occurrence, to determine the type of spawning [4, 21].

### 2.7. Estimation of the Gonadosomatic Index, the Condition Factor, and Spawning Period

The gonadosomatic index (GSI) based on wet weights was calculated using the formula of [22]: GSI = weight of ovary (g)/body weight of fish (g) − weight of gonads (g) × 100. Condition factor (CF) was calculated as CF = Wt/Lt^3^ × 10^5^ [23]. Reproductive period was determined by the temporal relative frequency distribution of the different stages of ovarian maturation [23].

### 2.8. Statistical Analyses

Distributions of raw data were checked for normality. The chi-square test (*χ* ^2^) was applied to check for possible differences between the proportions established. Student *t*-test was used to verify the differences between the total length and body mass of males and females. Pearson's correlation *t*-test was used to verify the relationships between the fecundity and total weight, fecundity and weight of gonads, and also between GSI and *K*. A significance at 5% level was adopted for all tests. 

## 3. Results

### 3.1. Sample Size, Length, and Body Mass

**Figure 1:**
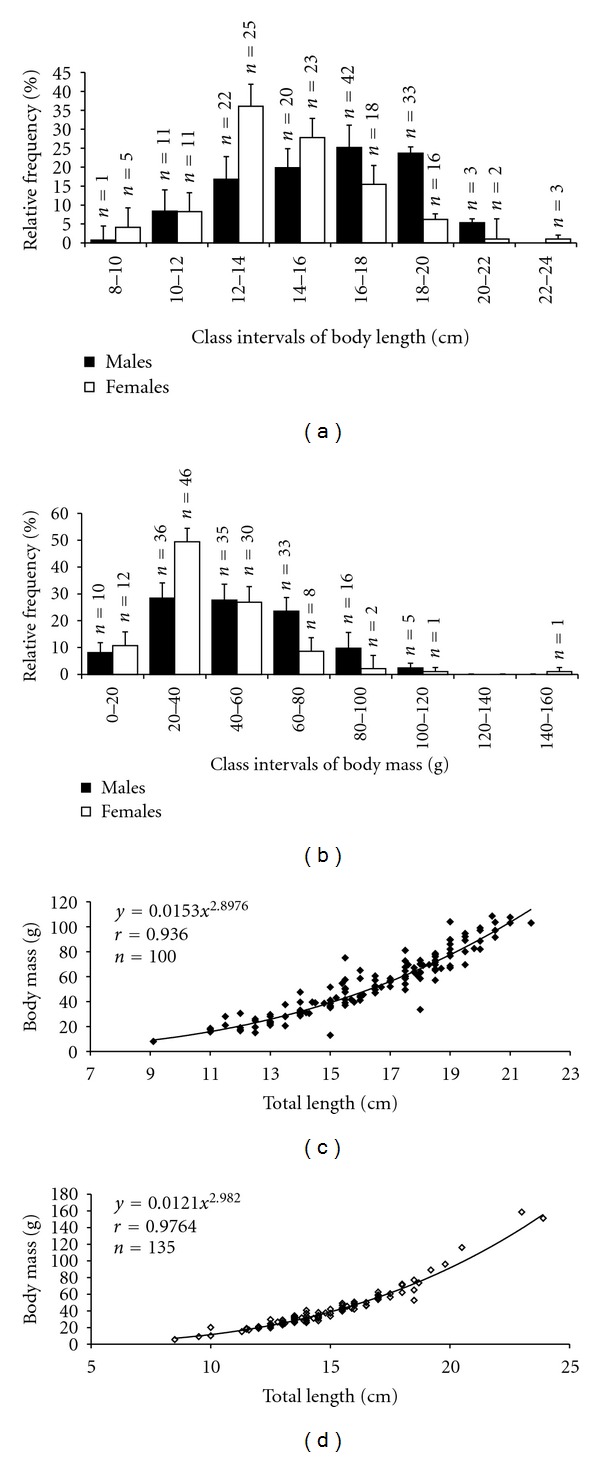
(a) Mean monthly distribution according to class intervals of total body length; (b) mean monthly distribution according to class intervals of body mass; (c) adjusted curve of empirical points of body mass and total body length of females; (d) adjusted curve of empirical points of body mass and total body length of males of *C.  menezesi*.

 A total of 235 samples of *C*.  *menezesi* were captured during the study period. Amplitude of total body length of males over the whole year varied from 12.64 cm to 17.2 cm, with an average of 16.2 cm (±SD 2.69). The same of females varied from 11.38 cm to 19.5 cm, with an average of 14.8 cm (±2.70). A higher frequency of occurrence of males in the class intervals 16 to 18 cm and the females in 12 to 14 cm was observed throughout the year (Figure 1(a)). The males were larger than the females with a significant statistical difference (*t* = 3.8004; *P* = 0.0002). 

The body mass of males varied from 32.4 g to 79.22 g (±13.64), with an average of 54.14 g (±2.45). The same of females varied from 24.8 g to 92.5 g, with an average of 39.72 g (±2.53). A higher frequency of occurrence of males and females in the class intervals of 20 to 40 g was observed (Figure 1(b)). There was a significant difference in the body mass of males and females (*t* = 2.41; *P* = 0.0246). The relationship between total weight (Wt) and total length (Lt) of *C.  menezesi* was positive for both sexes, indicating that this species increases in weight when there is an increase in length (Figures 1(c) and 1(d)). 

### 3.2. Sex Ratio

The frequency of occurrence of males (*n* = 135) and females (*n* = 100) of *C.  menezesi* indicated that the sex ratio was 1.3 M : 1 F, with a slight predominance of males (57.93%) in relation to females (42.07%). However, this difference was not statistically significant at 5% level (*χ* ^2^ = 2.51). There were alterations in the monthly distribution of both sexes, with more males during the months of March, July to December 2009, and January to February 2010. More females were registered during the months of April to June 2010. 

### 3.3. Body Length at First Gonadal Maturity (*L_50_*)

**Figure 2:**
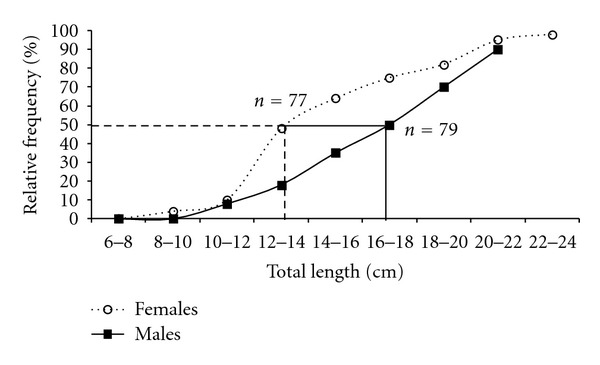
Relative frequency of first gonadal maturation (*L*_50_) of *C.  menezesi*.

The smallest mature female observed was 9.5 cm in length weighing 24.5 g, while the smallest mature male was 12 cm in length weighing 19 g. First gonadal maturity of females occurred at 13.8 cm of total length and that of males at 17.5 cm (Figure 2). The females of *C.  menezesi* attained sexual maturity earlier than the males.

### 3.4. Macroscopic Aspects of Ovaries and Testes

 The ovaries and testes were paired bilobed structures, symmetrical, elongated and joint in the posterior part to form a short duct leading to the urogenital pore. They were located in the posterior-dorsal part of the coelomic cavity, ventral to the kidneys and swim bladder. 

**Figure 3:**
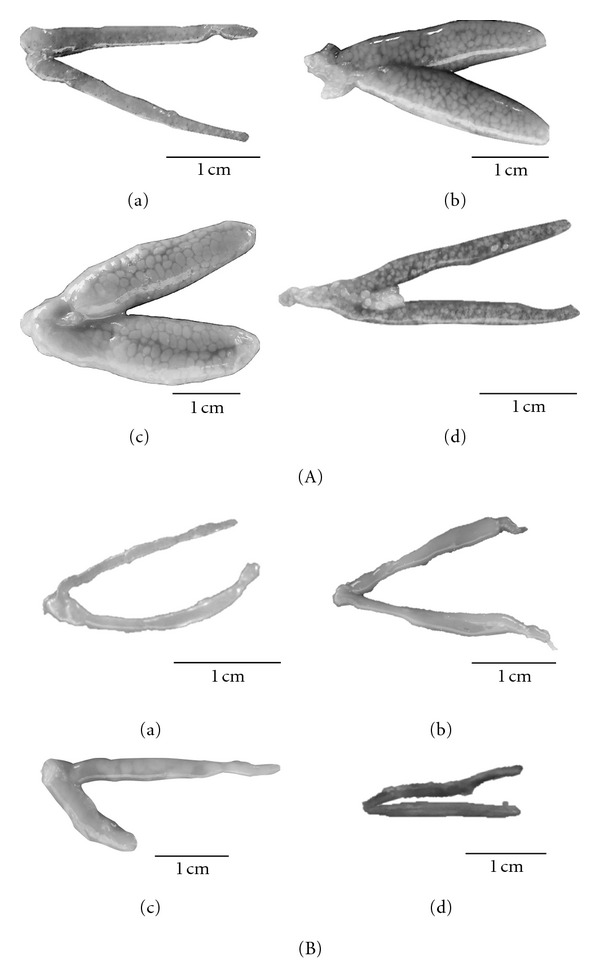
(A) Maturity stages of ovaries. (B) Maturity stages of testicles of *C*.  *menezesi*: (a) immature; (b) maturing; (c) mature; (d) partially spent (scale = 1 cm).

The macroscopic staging of the gonads based on the external morphology showed four stages of development: immature, maturing, mature, and partially spent. Stage I-immature: immature ovaries were very small, translucent, ribbon-like: occupying less than 1/3 of the coelomic cavity, with superficial vascularization. Immature testes were filamentous and translucent (Figure 3(a)). Stage II-maturing: the ovaries were small to medium in size, occupying about 1/3 to 2/3 of the coelomic cavity, with intense vascularization, and with small oocytes visible to the naked eye; maturing testes were fillet-like and were slightly whitish in colour (Figure 3(b)); Stage III-mature: the ovaries were large and turgid, orangish in colour, occupying about 2/3 to almost the entire coelomic cavity, with large visible oocytes; mature testes were turgid and whitish in colour (Figure 3(c)). Stage IV-partially spent: the ovaries and testicles were somewhat flaccid, with hemorrhagic appearance occupying less than half of the coelomic cavity (Figure 3(d)).

### 3.5. Histological Aspects of Ovaries

 Microscopic examination of ovarian sections showed that the oocyte development was consistent along the whole length of the ovary depending on the degree of maturation. Ovaries revealed four stages of ovarian development with five phases of oocyte development.

#### 3.5.1. Immature Stage

**Phase I-Chromatin Nucleolar Phase**

During the first growth stage, clusters of very small oogonia were found lying just beneath the ovigerous lamella. The young germ cells compactly filled the ovaries of young females.

**Phase II-Perinucleolar Phase**

**Figure 4:**
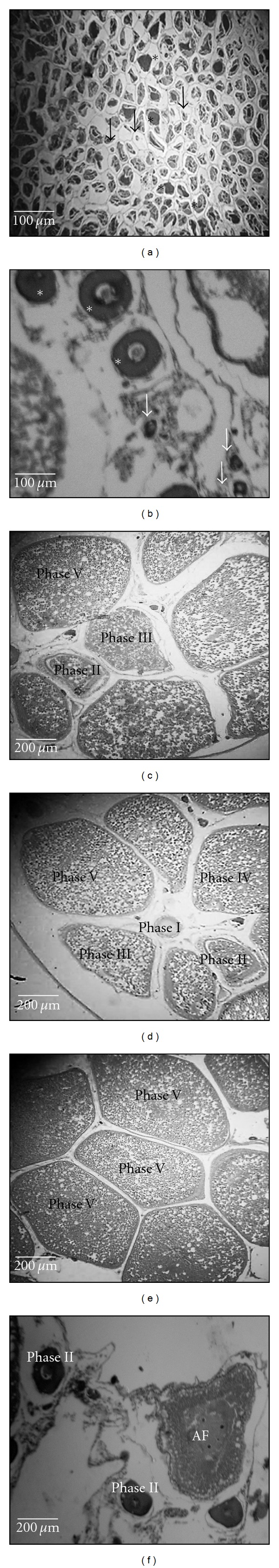
Histological aspects of oocytes developmental stages of *C*.  *menezesi*: (a) and (b) immature ovary showing oocytes with chromatin nucleolus (Phase I) arrows; chromatin perinucleolus oocytes (Phase II) ∗; (c) maturing ovary with reserve stock perinucleolus oocytes (Phase II), lipid vitellogenic stage oocytes (Phase III), lipid and protein vitellogenic stage oocytes (Phase IV); (d) and (e) mature ovary showing several batches of oocytes at different stages of development (Phase I to V); (f) partially spent stage showing oocytes phase III and atretic follicles (AFs).

The oocytes at this stage showed various forms as they developed, either round, triangular, rectangular, or oval. The oocytes present a large nucleus with multiple nucleoli distributed around the inner part of the nuclear envelope with a basophilic cytoplasm (Figure 4(a)).

#### 3.5.2. Maturing Stage

This stage was characterized by the presence of reserve stock oocytes (Phase II), oocytes with lipid droplets and cortical alveoli (Phase III), and oocytes with lipid droplets and protein (Phase IV) (Figure 4(b)).

**Phase III-Cortical Alveoli Phase**

This phase was characterized by the presence of cortical alveoli in the periphery. The nucleus had an irregular contour, and the cytoplasm showed presence of lipid droplets. The follicular layer showed an external surface with cells flatter than the theca. In this stage, the zona radiata appeared, surrounded by a layer of goblet cells and a layer of floor cells, comprising the granulose and theca, respectively.

**Phase IV-Early Yolk Phase**

Oocytes increased in size with the deposition of yolk, and the cytoplasm was filled with yolk granules. The nucleus was still in the center with an irregular outer surface. The follicle was composed of the zona radiata, followed by the granulose in a single layer of cells.

#### 3.5.3. Mature Stage

Mature oocytes (Phase V) appeared with yolk granules fused into yolk plates and oil droplets. The yolk granules gave the cytoplasm a grainy appearance. Migration of the nucleus to the micropylar end was observed. The mature oocytes were transformed from rounded to polyhedral in shape as a result of a compression effect. In addition to mature oocytes in large quantity (Phase V), various other phases of oocytes in development (Phases I to IV) were also present. Thus, mature ovaries showed the simultaneous occurrence of oocytes in different phases of development (Figure 4(c)). Partially spent stage: The ovaries of partially spent females showed hemorrhaging areas, empty spaces, and oocytes in maturation stage. Presence of postovulatory follicles, residual oocytes in the reabsorbing process of atresia, immature and maturing oocytes characterized this stage. Atretic oocytes were recognized by their irregular shape, disintegration of the nucleus, and liquefaction of the yolk granules (Figure 4(d)).

### 3.6. Histological Aspects of Testes

 The germinal epithelium of the testes was composed of four types of germ cells: spermatogonia, spermatocytes, spermatids, and spermatozoa. On the basis of histological changes, the testicular development was divided into four stages: immature, maturing, mature, and partially spent. 

#### 3.6.1. Immature Stage

**Figure 5:**
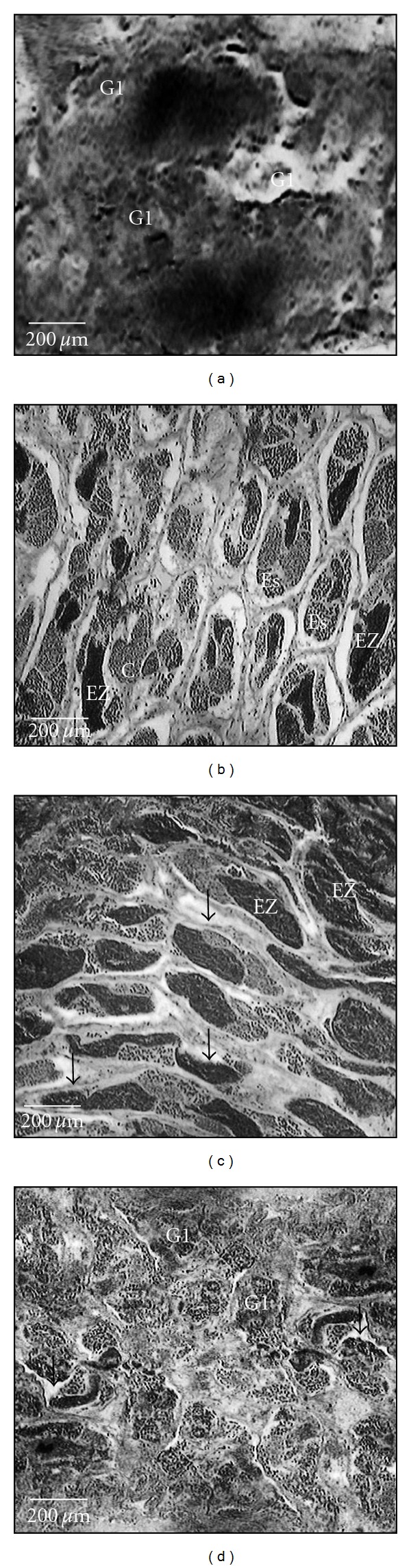
Histological aspects of testicles of *C*.  *menezesi* showing different stages of development: (a) immature testes with primary spermatogonia (G1); (b) maturing testes with spermatocytes (C); spermatids (Es) and spermatozoa (EZ); (c) mature testes with spermatozoa (Ez) and seminiferous tubules-arrows; (d) partially spent stage with primary spermatogonia and seminiferous tubules-arrows.

Spermatogonia were predominant (Figure 5(a)). There were primary and secondary spermatogonia. The primary spermatogonia were large with abundant cytoplasm and a large spherical nucleus with a nucleolus. They occurred in cysts in the seminiferous tubules during all stages of maturation. The secondary spermatogonia were smaller in size with dark nuclei.

#### 3.6.2. Maturing Stage

Primary and secondary spermatogonia, spermatocytes, and spermatids were present along the seminiferous tubules. During the latter part of this stage, small quantity of spermatozoa was observed (Figure 5(b)). The spermatocytes were spherical in shape, smaller than the spermatogonia, with a large nucleus and chromatin granules. The spermatids had spherical nuclei and were observed in cysts. 

#### 3.6.3. Mature Stage

The lobules were large with highly vascularized seminiferous tubules, which were packed with small-sized spermatozoa (Figure 5(c)). 

#### 3.6.4. Partially Spent Stage

The seminiferous tubules were empty with residual germ cells. Spermatogonia were present in sufficient quantities in the seminiferous tubules (Figure 5(d)).

### 3.7. Fecundity and Type of Spawning

**Figure 6:**
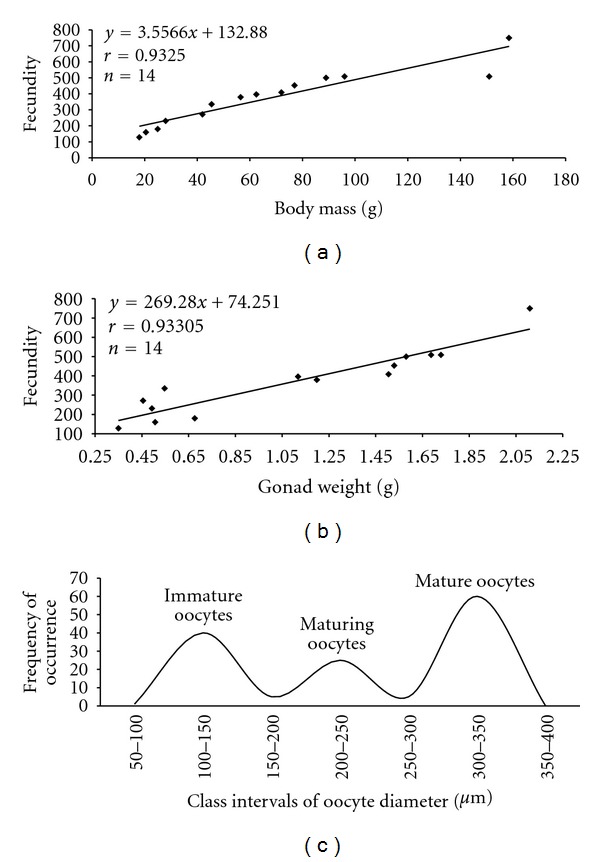
(a) Relationship between fecundity and body mass; (b) relationship between fecundity and weight of gonads; (c) frequency of occurrence of oocytes of different class intervals of mature *C*.  *menezesi*.

Fecundity and total body length of the female showed an exponential type of relationship (*r* = 0.913). There was linear relation between fecundity and body weight (*r* = 0.9325), and with fecundity and weight of gonads (*r* = 0.933), with positive correlations. The fecundity increased with the increase of length, body weight, and gonad weight (Figures 6(a) and 6(b)). The fecundity showed an amplitude of variation in the number of vitellogenic oocytes, ranging between 128 (±1.41) and 749 (±31.81) with a mean of 372 (±10.41) mature oocytes.

The distribution of the absolute frequency of oocytes with diameters of 50 *μ*m intervals indicated a synchronic development in more than two batches. Mature ovaries had several batches of oocytes at different stages of development, indicating partial spawning (Figure 6(c)).

### 3.8. Frequency of Occurrence of Gonadal Maturation Stages

**Figure 7:**
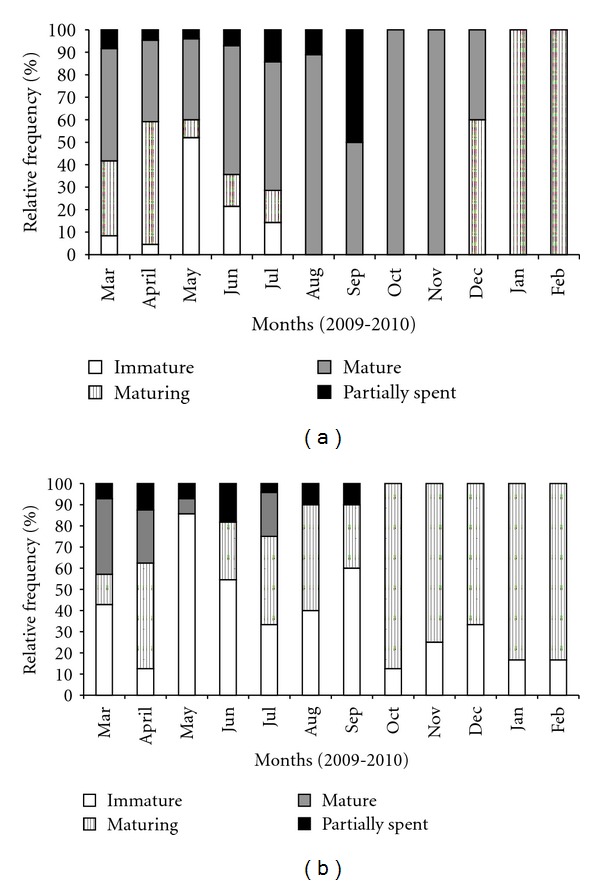
Monthly frequency of occurrence of gonadal maturity stages of *C*.  *menezesi* (a) females and (b) males.

Monthly frequency of ovarian maturation stages of *C*.  *menezesi* showed that mature individuals were predominant during almost all the months, especially from August to November, but were absent in January and February. Maturing females occurred from March to July and from December to February during the whole sampling period. Immature females occurred from March to July, and partially spent females occurred in June (Figure 7(a)). There was a predominance of immature and maturing males throughout the sampling period, but maturing males were not observed in May. Mature males occurred from March to May and in July. Partially spent males occurred in June (Figure 7(b)). 

### 3.9. GSI, *K*, and Reproductive Period

**Figure 8:**
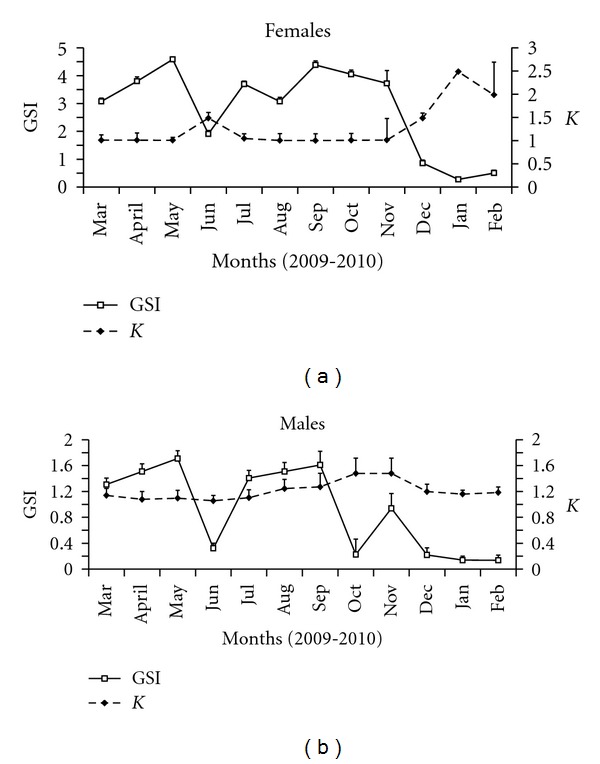
(a) Relationship between the GSI and *K* of *C.  menezesi* (a) females and (b) males.

The GSI values of females varied from 0.273 (±0.01) to 4.584 (±0.06), with an average of 2.448 (±0.74). Females had higher GSI during March to May and from September to November (Figure 8(a)). The GSI values of males varied from 0.136 (±0.08) to 1.308 (±0.10), with an average of 0.9192 (±0.11). Males had higher GSI during March to May and from July to September (Figure 8(b)). 

Condition factor (*K*) of females varied from 1.002 ± 0.08 to 2.485 ± 0.01, with an average of 1.045 ± 0.075 (Figure 8(a)), and that of males from 1.05 ± 0.08 to 1.478 ± 0.23, with an average of 1.207 ± 0.14 (Figure 8(b)). GSI and *K* were inversely correlated for both males (*r* = −0.9812; *P* < 0.05) and females (*r* = −0.9911; *P* < 0.05). 

*C*.  *menezesi* presented an extended reproductive period from March to September with peak periods from March to May and from July to September, based on the distribution of relative frequencies (%) of the maturity stages of the gonads and the variation of monthly GSI and *K*. 

## 4. Discussion

Cichlids have undergone spectacular adaptive radiation in the African and Neotropical regions. Adaptive changes in response to the varying ecological conditions, sexual and social selection, in addition to behavioral attributes, parental care and competition for territories and mates act as creative forces contributing to the outstandingly splendid speciation of cichlids [2, 24–26]. 

*Crenicichla menezesi* is endemic to the Caatinga ecoregion [9, 10]. The males of *C*.  *menezesi* are larger than the females, and the length-weight relationship in both sexes of this species shows isometric growth [11]. Generally, male cichlids are larger and heavier than females, as a consequence of behavioral evolution in pair formation and parental care, which increases their reproductive success [5, 15, 25, 27–29].

Some Neotropical cichlids like *Cichla monoculus* show sexual dimorphism, wherein males exhibit a postoccipital cephalic hump during the reproductive period with lipid accumulation [28]. In *Apistogramma cacatuoides*, sexual dimorphism is exhibited by males above 3 cm of body length, with prolonged filaments of the dorsal and caudal fins, whereas the females have truncated fins [30]. The study species *C*.  *menezesi* exhibited sexual dimorphism during the breeding phase. The females could be easily identified from the males, as they showed red coloration in the ventral region with lesser white dots on the body, thus the red color was highly intensified. This probably served to attract the males.

The study species presented a sex ratio within the expected 1 : 1 ratio. However, there was a slight predominance of males though not insignificant. Spatial sexual segregation may occur in the environment with males and females inhabiting different areas, which could explain the sex ratio observed in this study. Besides, the fishing gear utilized could have also contributed to this slight disparity [20, 31, 32].

The females of *C*.  *menezesi* attain sexual maturity earlier than the males, mobilizing their energy reserves for reproductive process well before the males. In the West African cichlid *Chromidotilapia guntheri*, both sexes attain sexual maturity with equal size, reflecting equal energy allocation to reproduction [33]. However, the time of sexual maturation is closely associated with body size of fish when the optimal size is reached [34]. 

This study describes the macroscopic and histological analysis of gonads of *C*.  *menezesi* which is important for a clear understanding of the reproductive process [35]. There is a regular pattern of development of the ovaries and testes of *C*.  *menezesi* during the different maturity stages, such as the immature, maturing, mature, and partially spent stages. These results are consistent with studies of other Neotropical cichlids, such as the Angel fish, *Pterophyllum scalare* [36], the peacock bass, *Cichla monoculus* [28], the Amazonian red discus, *Symphysodon discus* [4], and Cará, *Cichlasoma orientale* [37].

 The microscopic ovarian stages of the study species are typical of a multiple spawner. *C*.  *menezesi* spawns only a small portion of the oocytes during each breeding event. The concurrent presence of oocytes in all developmental stages in all fully developed and partially spent ovaries indicated asynchronous oocyte development in this species. Since the histological analyses do not represent oocytes of different-size diameters in a balanced proportion, wherein larger diameter oocytes are over represented, oocyte diameter distributions could be based on whole oocyte samples [21]. The incidence and duration of macroscopic ovarian stages of mature individuals of *C*.  *menezesi* also indicate a multiple spawning pattern. 

Generally, the testes are less studied than the ovaries, since females are considered more representative for the spawning season. The testes of mature and partially spent *C*.  *menezesi* showed the simultaneous occurrence of spermatozoa, spermatids, and spermatogonia, indicating that this species breeds more than once within a cycle. Similar results were observed for the cichlid red discus, *Symphysodon discus* [4, 38].

The oocyte development and fecundity pattern of *C*.  *menezesi* were characteristic of multiple spawners who release mature oocytes in batches [4, 21, 31]. In cichlids, fecundity is highly variable. The average fecundity of *C*.  *menezesi* is low, which is characteristic of species that exhibit extended reproductive period with parental care [28, 32]. Fecundity increases with increasing body size in *C*.  *menezesi*. In fish, fecundity is positively correlated with the body size due to energy availability for oocyte production [39]. The positive correlation between fecundity and body mass demonstrates that energy expenditure is involved in oocyte production [40, 41]. 

Most species of cichlids spawn up to four times during the reproductive cycle [25]. Partial spawning extends over a prolonged period, thus reducing competition among larvae, and the juveniles. The large reserve of yolk in oocytes allows larger and more developed larvae which are defended from predators by their parents, thus ensuring a higher survival rate of offspring and greater reproductive success [31].

The IGS and condition factor of *C*.  *menezesi* were negatively correlated. The low values of *K* may be related to the postreproductive stage, since there is a reduction of their metabolic activity and the consequent decline in feeding activity [31, 42]. An earlier study has demonstrated that there is a positive correlation between the accumulation of body lipids and the condition factor of the fish [40]. The body reserves are usually mobilized during the gonadal development and hence IGS and condition factor are negatively correlated, as observed in the study species.

 The IGS is useful to identify the reproductive period of total spawners or the peaks of reproductive activity in partial spawners. However, in addition to IGS, it is necessary to include data from histological analyses of gonads to correctly interpret the spawning period [43]. In the present study, both GSI and histological analyses of gonads were used to confirm the peaks of reproductive activity and the type of spawning. Due to the multiple spawning process of *C.  menezesi*,  the GSI reflected the peaks of reproductive activity during the study period.

This species is a partial spawner with an extended spawning period. The partial spawning process could be considered as an inner cycle, which is composed of ovulation, formation of mature oocytes, and spawning. This inner cycle continues throughout the reproductive season. This process occurs within the broad reproductive cycle of the fish [21]. A similar trend occurs in *C.  menezesi*. Based on the results of this study, it could be concluded that this species exhibits sexual dimorphism during the spawning season and females mature earlier than males. Both macroscopic and histological investigations of ovaries and testes revealed four stages of gonadal maturation and confirmed multiple spawning.

## 5. Conclusions

This study confirms that the males of *C.  menezesi* are larger, heavier, with a slight predominance than females, and mature before them. Both macroscopic and histological analyses show that males and females have four stages of gonadal development. This species present low fecundity with partial spawning over an extended period and is well adapted to the environmental characteristics of the semiarid climate of northeastern Brazil.
